# Development of Mouse Embryonic Stem (ES) Cells: IV Differentiation to Mature T and B Lymphocytes after Implantation of Embryoid Bodies Into Nude Mice

**DOI:** 10.1155/1995/52962

**Published:** 1995

**Authors:** Una Chen, Hoyan Mok

**Affiliations:** 1 Basel Institute for Immunology Grenzacherstrasser 487 Basel CH-4005 Switzerland; 2 Centre d'Immunologie de Marseille-Luminy Case 906 Marseille Cedex 9 F-13288 France

**Keywords:** ES fetuses, T and B lymphocytes

## Abstract

Mouse embryonic stem (ES) cells in culture can differentiate into late stages of many lineage-committed precursor cells. Under appropriate organ-culture conditions, ES cels differentiate into lymphoidlike cells at a stage equivalent to lymphoid cells found in fetal liver. These hematopoietic precursors are located in cup-shaped structures found in some embryoid bodies; we called such embryoid bodies “ES fetuses.” In this study, we have followed the maturation of hematopoietic cells after implantation of ES fetuses into nude mice for 3 weeks. ES-cell-derived lymphoid cells-pre-B cells, mature B cells, and mature T cells were found in all lymphoid organs. Interestingly, there was also an increase of T cells of host origin. Because native nude mouse lack thymus, these T cells might be educated by thymuslike epithelium generated from ES fetuses. Practical applications of this combined *in vitro* and *in vivo* system are discussed.


*Developmental Immunology*, 1995, Vol 4, pp. 79–84
Reprints available directly from the publisher
Photocopying permitted by license only

© 1995 Harwood Academic Publishers GmbH
Printed in Singapore

### Development of Mouse Embryonic Stem (ES) Cells: IV Differentiation to Mature T and B Lymphocytes after Implantation of Embryoid Bodies into Nude Mice

UNA CHEN* and HOYAN MOK

*Basel Institute for Immunology, Grenzacherstrasser 487, CH-4005 Basel, Switzerland*

Mouse embryonic stem (ES) cells in culture can differentiate into late stages of many lineage-committed precursor cells. Under appropriate organ-culture conditions, ES cells differentiate into lymphoidlike cells at a stage equivalent to lymphoid cells found in fetal liver. These hematopoietic precursors are located in cup-shaped structures found in some embryoid bodies; we called such embryoid bodies "ES fetuses." In this study, we have followed the maturation of hematopoietic cells after implantation of ES fetuses into nude mice for 3 weeks. ES-cell-derived lymphoid cells–pre-B cells, mature B cells, and mature T cells were found in all lymphoid organs. Interestingly, there was also an increase of T cells of host origin. Because native nude mouse lack thymus, these T cells might be educated by thymuslike epithelium generated from ES fetuses. Practical applications of this combined *in vitro* and *in vivo* system are discussed.

KEYWORDS: ES fetuses, T and B lymphocytes.

### INTRODUCTION

Mouse embryonic stem (ES) cells in culture can differentiate into precursor cells committed to many lineages (Martin, 1981; Doetschman et al., 1985; Robertson, 1987). We have previously defined culture conditions favorable for the differentiation of ES cells into hematopoietic cells including lymphoidlike cells at a stage equivalent to those found in fetal liver at days 10 to 14 after fertilization. These hematopoietic precursors are located in cup-shaped structures found in some embryoid bodies; we call such embryoid bodies "ES fetuses." Furthermore, using retroviruses and lymphokines, we established cell lines containing mixed populations of hematopoietic precursor cells (Chen, 1992). Some of these transformed cells were able to differentiate into mature lymphoid and myeloid cells after injection into sublethally irradiated F1 mice (Chen et al., 1992).

When single-cell suspensions obtained by mechanical disruption of ES fetuses were injected into immuno-incompetent SCID mice, the mice died within 2 days. Intraperitoneal injection led to the migration of cells, mainly to liver, and ES-derived lymphoid precursor cells differentiated into IgM secreting cells in a limiting-dilution pre-B-cell assay. When one or two whole embryoid boides were implanted under the kidney capsule of athymic nude mice, some mature lymphoid cells were found in the circulation as well as *in situ*. These preliminary observations suggested that ES-cell-derived hematopoietic precursors require an embryonic microenvironment for proper development in order to mature before they can migrate to primary and peripheral lymphoid organs in adult mice.

We report here that after implantation of ES fetuses under the skin of athymic nude mice, untransformed, mature B and T cells, as well as myeloid cells, of donor origin are found in the host. Moreover, the number of host T cells also increased, implying that the implanted embryoid bodies provide some thymic function.

* Corresponding author. Present address: Centre d'Immunologie de Marseille-Luminy, Case 906, F-13288 Marseille Cedex 9, France.

### RESULTS

### B Lymphocytes of Donor and Host Origin are Present in Chimeric Nude Mice

To document B-lymphocyte differentiation, ES fetuses were implanted subcutaneously into C57Bl/6 nude mice. Host and donor B cells can be distinguished by an allotypic difference of surface IgM. Three weeks after implantation, single-cell suspensions from bone marrow, lymph nodes, and spleen of chimeric mice were stained with FITC-labeled monoclonal antibodies (mAbs) specific for the $\mu^a$ and $\mu^b$ allotypes of B cells derived from donor and host, respectively. The proportion of cells positive for each surface marker, as well as the proportion of total sIg$^+$B cells, in each organ was estimated by flow cytometry. Figure 1 shows representative results of the one out of three experiments. The percentages of host-derived, donor-derived, and total B cells are summarized in Table 1.

ES-cell-derived $\mu^a$ positive B cells were found in bone marrow (BM), lymph nodes (LN), and spleen (Table 1). The percentage of donor B cells detected was highly variable; part of this variability may be related to the particular ES-cell line used as well as to the time of culture. Although there were fewer host B cells in the periphery than in control C57Bl/6 nude mice, the level in bone marrow was comparable. In all locations, the level of total sIg$^+$ B cells of chimeric mice was equal to or higher than the level of sIg$^+$ B cells in control nude mice.

In the chimeric nude mice experiments, we found that the degree of chimerism, that is, the ratio of mature donor ES-cell-derived B cells to host B cells changes as a function of the length of culture *in vitro*. In preliminary experiments, we established that no donor B cells could be detected when the ES fetuses were implanted earlier than 14 days or longer than 31 days (data not shown). The ratio of donor to host $\mu^+$ B cells was less than 1 when the ES

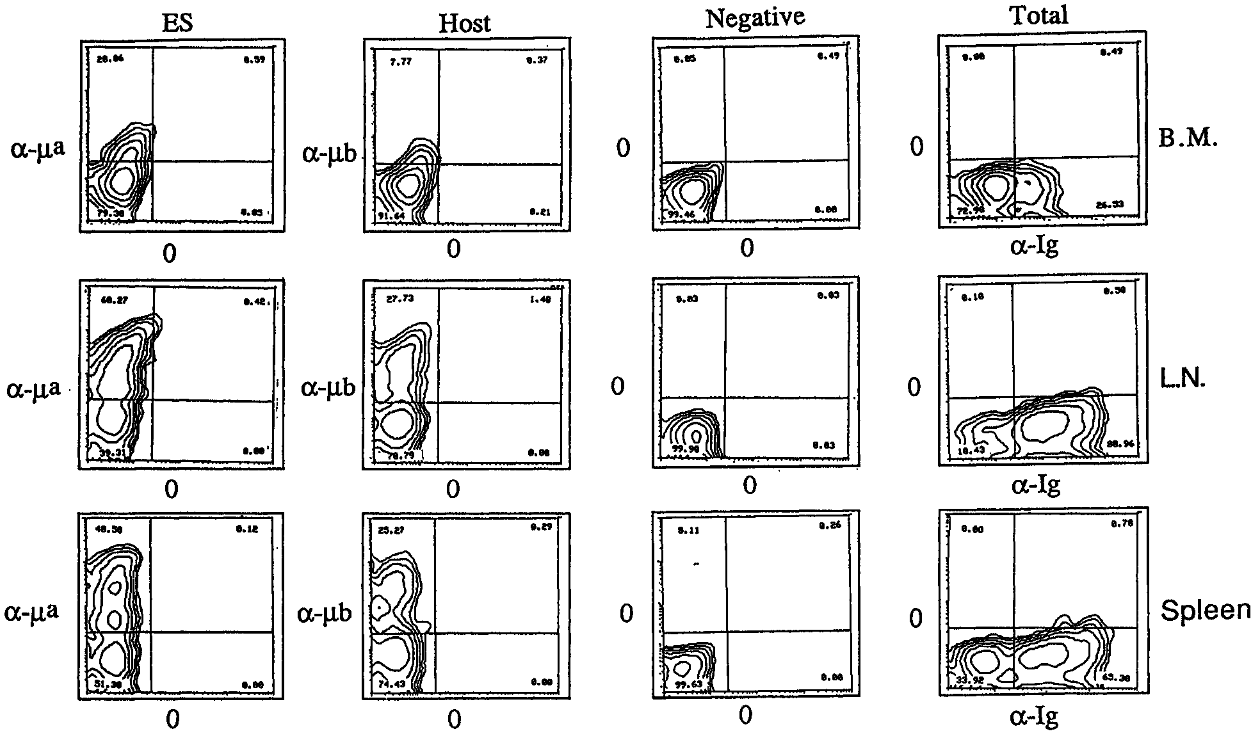

FIGURE 1. Flow cytometric analysis of B cells derived from ES cells (-$\mu^a$), host (-$\mu^b$), control (negative), or total (sIg) B cells from bone marrow (BM), lymph nodes (LN), or spleen. The number in each of the four compartments indicates the percentage of cells of each individual staining mAb, as indicated on the $x$-axis and $y$-axis of ech contour figure. Biotin-labeled [a] (Sieckmann et al., 1991), biotin-labeled $\mu^b$ (Stall and Loken, 1984), and PE-labeled streptavidin; sIg, FITC-labeled sheep anti-mouse Ig heavy and light chains, $F(ab)'_2$ fragment (Silenus, Hawthorotreet, Australia). $1 \times 10^6$ cells per mAb staining was performed and a minimum of $1 \times 10^4$ cells were analyzed per sample.

TABLE 1
B cells of donor and host origin in chimeric mice*

| Expt. no. | Origin/ conditions | Organ | $^{a*}$ (donor)† | $^{b}$ (host) | Ratio $^{a}/^{b}$ | $\delta^a$ (donor) | $\delta^b$ (host) | ratio $\delta^a/\delta^b$ | Total sIg | B220 | Negative control |
|---|---|---|---|---|---|---|---|---|---|---|---|
| I-1 | ES fetuses of line D3 | BM | 0.4 | 0.7 | 0.5 | 5.5 | 3.4 | 1.6 | 10.2 | 3.7 | 0.1 |
| | cultured for 20 days | LN | 10.4 | 36.8 | 0.3 | 13.5 | 45.9 | 0.3 | 72.0 | 72.5 | 0.2 |
| | | Spleen | 2.9 | 29.3 | 0.1 | 11.9 | 33.3 | 0.4 | 48.8 | 50.9 | 0.3 |
| 1–2 | BM cells from Expt. I-1 | BM | 1.1 | 0.3 | 3.5 | n.d. | 0.2 | n.d. | 4.0 | 4.7 | 0.3 |
| | $5 \times 10^6$ cells/mouse, iv | LN | 61.4 | 2.3 | 26.3 | n.d. | 2.2 | n.d. | 90.8 | 95.1 | 0.1 |
| | | Spleen | 46.5 | 1.7 | 27.5 | n.d. | 2.2 | n.d. | 52.5 | 55.2 | 0.9 |
| II | ES fetuses of line D3 | BM | 1.0 | 1.4 | 0.7 | 11.6 | 3.6 | 3.2 | 2.8 | 4.7 | 0.3 |
| | cultured for 27 days | LN | 53.1 | 1.7 | 30.5 | 88.2 | 1.1 | 80.1 | 86.6 | 90.7 | 0.1 |
| | | Spleen | 26.3 | 0.3 | 87.1 | 31.2 | 1.7 | 18.4 | 31.2 | 35.6 | 0.9 |
| III-1 | ES fetuses of line CCE | BM | 20.0 | 7.7 | 2.5 | 28.7 | 1.5 | 19.1 | 26.5 | 28.4 | 0.1 |
| | cultured for 20 days | LN | 60.2 | 27.7 | 2.1 | 63.4 | 24.6 | 2.6 | 89.0 | 90.0 | 0.1 |
| | ( = Fig. 1) | Spleen | 48.5 | 25.2 | 1.9 | 31.1 | 16.7 | 1.8 | 65.3 | 69.8 | 0.1 |
| III-2 | BM cells from Expt III-1 | BM | 3.2 | 2.3 | 1.4 | n.d. | 3.5 | n.d. | 13.3 | 9.1 | 0.4 |
| | $5 \times 10^6$ cells/mouse, iv | LN | 57.3 | 5.2 | 10.9 | n.d. | 6.0 | n.d. | 79.5 | 86.6 | 0.7 |
| | | Spleen | 51.2 | 3.9 | 12.9 | n.d. | 3.8 | n.d. | 69.5 | 70.5 | 1.1 |
| IV | C57B1/6 nu/nu mice | BM | 0.1 | 6.6 | 0.02 | 1.6 | 6.8 | 0.2 | 7.3 | 16.6 | 0.1 |
| | (control) | LN | 0.2 | 82.7 | <0.01 | 1.2 | 71.8 | <0.01 | 82.9 | 88.9 | 0.4 |
| | | Spleen | 0.1 | 72.9 | <0.01 | 1.0 | 85.2 | <0.01 | 80.4 | 89.3 | 0.3 |
| V | Strain 129 mice | BM | 1.9 | 0.2 | 9.5 | 1.0 | 0.4 | 6.3 | 2.5 | 2.8 | 0.1 |
| | (control) | LN | 47.7 | 3.0 | 15.9 | 49.8 | 2.4 | 20.8 | 52.7 | 55.6 | 1.0 |
| | | Spleen | 55.5 | 1.2 | 46.3 | 54.4 | 0.8 | 68.0 | 56.8 | 56.8 | 0.1 |

* ES fetuses were implantated into C57B1/6 nude mice; 3 weeks later cells were anlayzed by flow cytometry and bone marrow cells were transferred into secondary hosts, which were analyzed 3 weeks after cell transfer. All numbers are percent of nucleated cells in each organ.
† mAb $\mu^a$ (Stall and Loken, 1984), $\mu^b$ (Sieckman et al., 1991), $\delta^a$, $\delta^b$ (Stall and Loken, 1984), B220 (Coffman and Weissman, 1981; Kincade et al., 1981).
Negative control: Cells staining with secondary antibody only.

fetuses had been cultured for 20 days and increased 100-fold when the culture period was increased by 1 week of culture (Table 1, Experiments I and II). There were also even fewer host B cells in the periphery.

Interestingly, the reduction in peripheral, host B cells observed when long-term cultured ES fetuses were used as the source of cells was also observed when chimeric bone marrow cells from the first host were transferred to secondary hosts. After the transfer of chimeric bone marrow cells, ES-derived B cells migrated preferentially to lymph nodes and spleen of the second host, that is, the percentage of host B cells was much less than in unimplanted control mice. This type of reduction was observed with both ES-cell lines used, CCE line and D3 (Table 1).

### T Lymphocytes and Myeloid Cells of Donor and Host Origin Are Present in Chimeric Nude Mice

To document the differentiation of T lymphocytes and other cell types, ES fetuses were implanted subcutaneously into Balb/c nude mice. Host and donor cells can be distinguished by a haplotypic difference in surface MHC class I antigens. Three weeks after implantation, single-cell suspensions from bone marrow, lymph nodes, and spleen of chimeric mice were stained with FITC-labeled mAbs specific for the H-2K$^b$ and H-2D$^d$ antigens of cells derived from donor and host, respectively. In addition, cells from each experiment were analyzed for expression of several T-cell markers (CD4, CD8, $\alpha\beta$ TCR), B-cell markers (sIg, B220, and GP-76) as well as a marker of cells of the myeloid lineage (Mac-1). The proportion of cells positive with each surface marker in lymph nodes and spleen was estimated by flow cytometry. The percentages of cells positive for these markers among donor- and host-derived cells are summarized in Table 2.

In most experiments, sIg$^+$, B220$^+$ mature B cells were present, as well as GP-76 pre-B cells (Strasser, 1988). Mac-1$^+$ myeloid cells were also present in all experiments. A small but significant number of cells expressed CD4, CD8, and $\alpha\beta$ TCR T cells of both donor and host origin. In lymph nodes, we detected a few CD4$^+$8$^+$ double-positive T cells of donor origin (data not shown). These double-positive T cells most likely account for the total percentage of cells found in experiments in Table 2, exceeding the theoretical maximum value. Thus, precursor B, B, and T cells, as well as myeloid cells, developed in the chimeric mice.

TABLE 2
Lymphoid and Myeloid Cells of Donor and Host Origin in Chimeric Mice*

| Expt. no. | Origin/conditions/ anti-class I mAb† | Organ | GP-76 | sIg | B220 | Mac-1 | αβTCR | CD4 | CD8 |
|---|---|---|---|---|---|---|---|---|---|
| Ia | ES fetuses of line D3 | LN | 5.6 | 9.9 | 10.2 | 4.4 | 7.3 | 6.7 | 4.5 |
| | cultured for 19 days | Spleen | 6.1 | 6.5 | 5.4 | 6.0 | 6.5 | 2.2 | 1.3 |
| | anti-H-2K$^b$ (donor†) | | | | | | | | |
| Ib | ES fetuses of line D3 | LN | 30.1 | 63.1 | 54.1 | 13.2 | 13.4 | 11.7 | 8.5 |
| | cultured for 19 days | Spleen | 15.2 | 39.5 | 25.7 | 43.6 | 18.7 | 8.7 | 17.4 |
| | anti-H-2D$^d$ (host†) | | | | | | | | |
| IIa | Balb/c nu/nu mice | LN | 0.1 | 0.7 | 0.6 | 0.4 | 0.7 | 0.1 | 0.1 |
| | anti-H-2K$^b$ (control) | Spleen | 0.5 | 0.2 | 0.1 | 0.2 | 0.2 | 0.1 | 0.1 |
| IIb | Balb/c nu/nu mice | LN | n.d. | n.d. | n.d. | n.d. | n.d. | n.d. | n.d. |
| | anti-H-2D$^d$ (control) | Spleen | 0.5 | 56.3 | 56.6 | 3.1 | 9.5 | 2.0 | 2.3 |
| III | Strain 129 mice | LN | 7.3 | 45.6 | 50.6 | 1.6 | 46.5 | 40.0 | 8.1 |
| | anti-H-2K$^b$ (control) | Spleen | 1.9 | 50.7 | 54.4 | 2.6 | 25.3 | 32.2 | 7.6 |

* ES fetuses were implanted into Balb/c nude mice; three weeks later cells were analyzed by flow cytometry. All numbers are percent of nucleated cells in each organ.
† H-2 K$^b$: MHC class I of donor origin reacting with mAb B8-24-3 (Köhler et al. 1981); H-2D$^d$: MHC class I of host origin reacting with mAb 15.5.5 (Ozato et al., 1980). mAb GP-76 (Strasser, 1988), B220 (Coffman and Weissman, 1981; Kincade et al., 1981), Mac-1 (Springer et al., 1979), αβTCR (Kubo et al., 1989), CD4 (Wilde et al., 1983), CD8 (Ledbetter and Herzenberg, 1979).
n.d.: not done.

### DISCUSSION

It should be emphasized that the results to be reported here, like those reported earlier (Chen et al., 1992), can only be obtained when "ES fetuses"—embryoid bodies selected for the presence of the cup-shaped structures containing lymphocyte precursors—are used. For the present experiments, we have implanted the cultured ES fetuses subcutaneously rather than under the kidney capsule implantation because it is simpler and faster; the operation takes about 1 min. instead of 30 min. Moreover, the space for implantation of embryoid bodies is larger; 10 bodies can be implanted at one site instead of one or two. It is also likely that cell migration is easier without the capsule.

### Lymphocytes of Donor and Host Origin are Present in Chimeric Nude Mice

Depending on the strain combination of chimerism, we could follow the maturation and migration of B cells only in 129 (ES)-C57B1/6 chimeric nude mice or donor T and B cell in 129 (ES)-Balb/c chimeric mice. As summarized in Table 1, a high ratio of B cells of donor vs. host in the chimeric nude mice was detected in this study, and the total amount of sIg was comparable to the values found in control mice. This phenomenon could be due to any or all of the following: (a) ES-derived B cells proliferate faster than host B cells and thus take over the peripheral B-cell pool. (b) ES-derived cells were activated nonspecifically either in prolonged culture or in chimeric bone marrow so that they were primed to suppress host B cells. (c) Donor cells that actively suppress the host B cells may not be B cells or precursor B cells. The presence of mature T cells, shown in Table 2, implies thymic function of the implant to allow the education of mature CD4$^+$ and CD8$^+$ T cells (review by von Boehmer, 1990; von Boehmer and Rajewsky, 1993). Because there was a quantitative increase of T cells of host origin after ES fetus implantation, the implant seemed to also function as a thymus for extrinsic host cells.

### Quo Vadis?

As we were able to obtain cells of various lineages at relatively high frequencies after implantation of the cystic embryoid bodies that we call ES fetuses, it might be possible to also obtain untransformed, committed lineage-specific stem cells for many purposes. This would provide us with a way to dissect the development of lineage-specific stem cells. In addition, such a study could have practical applications in understanding some complications that occur during the repopulation of immunodeficient mice with transferred lymphocytes from embryonic sources.

Because the time needed for one experiment of *in vitro* formation of ES fetus and *in vivo* differentiation to mature lymphoid or myeloid cells is 7 weeks, the method described in this study may serve as a shortcut for studying the expression of lymphoid-specific genes in both embryonic and adult stages after mutation or transfection of genes in ES cells (Chen et al., manuscript in preparation).

### MATERIALS AND METHODS

### Culture of ES Cells and the Differentiation of Embryoid Bodies

Male ES cells (ES-D3) (Doetschman et al., 1985; Gossler et al., 1986) of strain 129 origin were a gift from Rolf Kemler, Max Planck Institute for Immunobiology, Freiburg, Germany. Male ES cells (ES-CCE) (Robertson, 1987) of strain 129 were a kind gift from Elizebeth Robertson and Fred Alt, Harvard University. Undifferentiated ES-cell lines were grown on embryonic feeder cells in DMEM supplemented with 15% heat-inactivated, preselected fetal calf serum. After reaching confluence, the ES cells were allowed to differentiate in a suspension culture for 36 days (Chen, 1992). Briefly, about $1\text{–}2 \times 10^5$ ES cells were inoculated into ES differentiation medium (i.e., DMEM, supplemented with 15% preselected, heat-inactivated FCS, $10^{-4}$ M 2-mercaptoethanol and antibiotics) without feeder cells into a 60×15 mm hydrophobic culture dish (Heraeus, Heusenstamm, Germany). The cultures were kept at 37°C in a humidified incubator with air and 5% $CO_2$ and the medium was changed daily for 36 days. Within 4–5 days, the cell clusters expanded three-dimensionally such that simple embryoid bodies could be observed. Complicated structures, for example, pulsating cardiac muscle, and yolk sac blood islands, developed inside some of the bodies after another 4–5 days. At this stage of development, the structures are called cystic embryoid bodies (Doetschman et al., 1985; Robertson, 1987). At the same time or slightly after the appearance of cardiac muscle and blood islands, some cystic embryoid bodies developed a cup-shaped structure containing lumphoidlike cells (Chen et al., 1992). Such ES fetuses were taken for implantation at day 11–12, day 17–18, day 25–26, and day 35–36 of culture.

### Implantation of ES Fetuses into Adult Mice

Four- to six-week-old male nu/nu C57B1/6 mice and nu/nu Balb/c mice were used as hosts. This was performed with mice under general anesthesia administered by intraperitoneal injection of Avertin (8 mg per 0.4 ml per 10 grams of body weight; Ardreich, Steinheim, Germany). Ten mice per group received implants subcutaneously in the lumbar region superficial to the spinal column using general surgical procedures. Three weeks post surgery, most of the mice were sacrificed. Organs and tissues were removed and prepared for cryostat sectioning and for flow cytometry assays (Chen and Kosco, 1993). For animal experiments, the experimental license number is Bewilligung-714 of the Basel Institute for Immunology.

### Characterization of Lymphoid and Myeloid Cell Populations by Flow Cytometry

In C57B1/6 chimeras, B cells of donor and host origins were distinguished by the IgM and IgD allotype expressed on the surface of B cells—$\mu^a$ and $\delta^a$, or $\mu^b$ and $\delta^b$, for donor and host, respectively. In Balb/c chimeras, lymphoid and myeloid populations of donor and host origin were distinguished by the MHC class I antigen—H-2$K^b$ and H-2$D^d$ for donor and host, respectively. The mAbs used were biotin-labeled B8-24-3 (anti-H-2$K^b$; Köhler et al., 1981) and Texas Red-labeled streptavidin (Southern Biotechnology, Birmingham), and biotin-labeled 15.5.5 (anti-H-2$D^d$; Ozato et al., 1980, 1985), and Texas Red-labeled streptavidin. In addition, FITC-labeled goat anti-mouse $\mu$-chain $F(ab)'_2$ (Silenus, Hawthorotreet, Australia). Secondary reagents included FITC-labeled rabbit anti-hamster IgG $F(ab)'_2$, sheep anti-mouse Ig (heavy and light chains) $F(ab)'_2$. Immunofluorescence was evaluated by flow cytometry. Gating of lymphocytes was performed according to the FACS Lysis II program as described in detail in the manual by Becton-Dickinson; a similar gating method has been previously used by Chen (1992). The standard deviation of values in Tables 1 and 2 was 2%.

### ACKNOWLEDGMENT

We appreciate helpful discussion and critical reading of this manuscript by Marie Kosco, Wayne Hein, Ed Palmer, Howard Etlinger, and particularly by Charles Steinberg. The Basel Institute for Immunology was founded and is supported by F. Hoffmann-La Roche, Basel, Switzerland. The first author was partially supported by an EC short-term fellowship.

*(Received May 10, 1994)*

*(Accepted October 31, 1994)*

### REFERENCES

Chen U. (1992). Differentiation of mouse embryonic stem cells to lympho-hematopoietic lineages in vitro. Dev. Immunol. **2**: 29–50.

Chen U., and Kosco M. (1993). Differentiation of mouse embryonic stem cells in vitro: III. Morphological evaluation of tissues developed after implantation of differentiated mouse embryoid bodies. Dev. Dynamics **197**: 217–226.

Chen U., Kosco M., and Staerz U. (1992). Establishment and characterization of lymphoid and myeloid mixed cell populations from mouse late embryoid bodies, "ES fetuses." Proc. Natl. Acad. Sci. USA **89**: 2541–2545.

Coffman R.L., and Weissman I.L. (1981). B220: A B cell-specific member of the T200 glycoprotein family. Nature **289**: 681–683.

Doetschman T., Eistetter H., Katz M., Schmidt W., and Kemler R. (1985). The in vitro development of blastocyst-derived embryonic stem cell lines: Formation of visceral yolk sac, blood islands and myocardium. J. Embryol. Exp. Morph. **87**: 27–45.

Gossler A., Doetschman T., Korn R., Serfling E., and Kemler R. (1986). Transgenesis by means of blastocyst-derived embryonic stem cell lines. Proc. Natl. Acad. Sci. USA **83**: 9065–9069.

Kincade P.W., Lee G., Watanabe T., Sun L., and Scheid M.P. (1981). Antigens displayed on murine B lymphocte precursors. J. Immunol. **127**: 2262–2268.

Köhler G., Fischer-Lindahl K., and Heusser C. (1981). Characterization of a monoclonal anti-H-$2K^b$ antibody. Steinberg C., and Lefkovits I., Eds. (Basel, Switzerland: Karger) Immune System **2**: 202–208.

Kubo R.T., Born W., Kappler J.W., Marrack P., and Pigeon M. (1989). Characterization of a monoclonal antibody which detects all murine $\beta$ T cell receptors. J. Immunol. **142**: 2737–2743.

Ledbetter J.A., and Herzenberg L.A. (1979). Xenogeneic monoclonal antibodies to mouse lymphoid differentiation antigens. Immunol. Rev. **47**: 63–75.

Martin G.R. (1981). Isolation of a pluripotent cell line from early mouse embryos cultured in medium conditioned by teratocarcinoma stem cells. Proc. Natl. Acad. Sci. USA **78**: 7634–7638.

Ozato K., Mayer N., and Sacks D. (1980). Hybridoma cell lines secreting monoclonal antibodies to mouse H-2 and Ia antigens. J. Immunol. **124**: 533–540.

Ozato K., Wan Y-J., and Orrison B.M. (1985). Mouse major histocompatibility class I gene expression begins at midsomite stage and is inducible in earlier-stage embryos by interferon. Proc. Natl. Acad. Sci. USA **82**: 2427–2431.

Robertson E.J. (1987). Teratocarcinomas and embryonic stem cells, a practical approach, Robertson E.J., Ed. (Oxford; Washington, DC: IRL Press).

Rocha B., Vassalli P., and Guy-Grand D. (1992). The extrathymic T-cell development pathway. Immunol. Today **13**: 449–451.

Sieckmann D.G., Stall A.M., and Subbaro B. (1991). A mouse monoclonal antibody specific for an allotypic determinant of the Igha allele of murine IgM: Genetic and functional analysis of Igh-6a epitopes using anti-IgM monoclonal antibodies. Hybridoma **10**: 121–135.

Springer T., Galfré G., Secher D.S., and Milstein C. (1979). Mac-1: A macrophage differentiation antigen identified by monoclonal antibody. Eur. J. Immunol. **9**: 301–306.

Stall A.M., and Loken M.R. (1984). Allotypic specificities of murine IgD and IgM recognized by monoclonal antibodies. J. Immunol. **132**: 787–795.

Strasser A. (1988), PB76: A noval surface glycoprotein preferentially expressed on mouse pre-B cells and plasma cells detected by the monoclonal antibody G-5-2, Eur. J. Immunol. **18**: 1803–1810.

Von Boehmer H. (1990). Developmental biology of T cells in T cell receptor transgenic mice. Annual Rev. Immunol. **8**: 531–556.

Von Boehmer H., and Rajewsky K. (1993). Lymphocyte development. Curr. Opin. Immunol. **5**: 175–177.

Wilde D.B., Marrack P., Kappler J., Dialynas D.P., and Fitch F.W. (1983). Evidence implicating L3T4 in class II MHC antigen reactivity; monoclonal antibody GK1.5 (anti-L3T4a) blocks class II MHC antigen-specific proliferation, release of lymphokines, and binding by cloned murine helper T lymphocyte lines. J. Immunol. **131**: 2178–2183.

